# Rates of discontinuation and non-publication of upper and lower extremity fracture clinical trials

**DOI:** 10.1186/s13018-023-03698-5

**Published:** 2023-03-29

**Authors:** Samuel Shepard, J. Michael Anderson, Benjamin Heigle, Jay C. Thompson, Byron Detweiler, Micah Hartwell, Matt Vassar

**Affiliations:** 1 Oklahoma State University Center for Health Sciences, 1111 W 17Th St, Tulsa, OK 74107 USA; 2 Department of Orthopedic Surgery, Oklahoma State University Medical Center, 744 West 9Th St, Tulsa, OK USA; 3 Department of Psychiatry and Behavioral Sciences, Oklahoma State University Center for Health Sciences, 1111 W 17Th St, Tulsa, OK 74107 USA

**Keywords:** Fracture, Upper extremity, Lower extremity, Non-publication, Discontinuation, Clinical trials

## Abstract

**Purpose:**

To our knowledge, no study has quantified the rate of discontinuation and nonpublication of randomized controlled trials (RCTs) regarding upper and lower extremity fractures.

**Methods:**

We searched ClinicalTrials.gov on September 9th, 2020, for phase 3 and 4 RCTs pertaining to upper and lower extremity fractures. Trial completion status was determined using records available on ClinicalTrials.gov. Publication status was determined using records on ClinicalTrials.gov and by searching PubMed (MEDLINE), Embase, and Google Scholar. We queried corresponding authors on trial status if a peer-reviewed publication was not identified.

**Results:**

Our final analysis included 142 RCTs, of which 57 (40.1%) were discontinued and 71 (50%) were unpublished. Thirty-six (of 57, 63.2%) discontinued trials failed to provide a reason for discontinuation, the most commonly identified reason for discontinuation was due to inadequate recruitment (13/21, 61.9%). Completed trials were more likely to reach publication (59/85; 69.4%; *X*^*2*^ = 32.92; *P* ≤ 0.001) than discontinued trials. Trials with more than 80 participants were less likely not to reach publication (AOR: 0.12; 95% CI 0.15–0.66).

**Conclusion:**

Our analysis of 142 upper and lower extremity fracture RCTs demonstrated one-half failed to reach publication and two-fifths were discontinued prior to trial completion. These findings indicate the need for increased guidance in developing, completing, and publishing RCTs in upper and lower extremity fractures. Discontinuation and nonpublication of orthopaedic RCTs hinder the public’s access to collected data and negate the valued contribution from study participants. Discontinuation and non-publication of clinical trials may subject participants to potentially harmful interventions, limit the advancement of clinical research, and contribute to research waste.

*Level of Evidence*: III.

**Supplementary Information:**

The online version contains supplementary material available at 10.1186/s13018-023-03698-5.

## Introduction

In the USA, the annual incidence of age-related fractures is expected to surpass 3 million fractures per year by 2025 [1]. More specifically, the incidence of upper and lower extremity fractures is estimated to occur at a rate of 67 and 70 per 100,000 people per year, respectively [2, 3]. By the year 2040 the projected cost for fracture care is expected to exceed $90 billion [4]. Given the high prevalence and significant medical expenses required to manage upper and lower extremity fractures, it is essential that health policy and clinical decision-making is guided by robust and reliable evidence.

Atop the evidence-based hierarchy are randomized controlled trials (RCTs), which are often regarded as Level-I evidence by some orthopaedic surgery journals [5]. Results from these studies are used to establish clinical recommendations, thereby directly influencing patient care. For example, the American Academy of Orthopedic Surgeons (AAOS) cites the results of an RCT as supporting evidence for a strong-grade (Level A) recommendation for the use of a multimodal pain regime to minimize narcotic associated delirium in patients following bipolar hemiarthroplasty for hip fractures [6, 7]. Considering the implications these studies have on clinical practice, it is critical that all results––regardless of nature or statistical significance––are readily accessible for both patients and clinicians, alike. However, previous research suggests that upwards of 85% of biomedical research is wasted––accounting for more than US $170 billion spent on potentially wasted research [8]. The exact etiology behind this burden of wasted research is likely multifactorial; however, two contributing factors are studies that fail to reach publication or studies that are erroneously discontinued.

Failing to publish research outcomes is not uncommon with current estimates suggesting up to one-half of RCTs never reach publication [9]. Failing to publish these outcomes may hinder scientific advancement. Publication bias––defined as the increased likelihood of a study with statistically significant results reaching publication––is a well-documented source of bias within medical research [10, 11]. Some contend that nonpublication of research findings jeopardizes the reliability and trust in scientific research [12, 13]. Others claim that nonpublication may result in weaker evidence and hinder any true association between a particular intervention and positive outcomes [13]. Similar to nonpublication, trial discontinuation is another mechanism by which resources are wasted.

Trial discontinuation may result from necessary circumstances, including patient safety, poor trial efficacy, or the feasibility of completion. However, many documented reasons for trial discontinuation may be preventable [14, 15]. According to the Declaration of Helsinki, trial discontinuation for personal or financial purposes is unethical, contributes to research waste, and may threaten the safety of research participants [16, 17]. From a global perspective, trial discontinuation for any reason may jeopardize patient–physician relationships in terms of missing out on accessing valuable research that may benefit treatment” [15]. As a similar study has already been conducted in otolaryngology [18], it has shown the importance of such an analysis in uncovering biases, improving the transparency and reliability of the evidence base, and ultimately advancing the field. Conducting this study in the field of upper and lower extremity fractures will allow us to contextualize our findings among existing literature and identify unique challenges faced in this field. Moreover, this study will call attention to areas for improvement in the clinical trial process, including increased transparency and ethical considerations, and provide recommendations for future trials in this field. By conducting this study, we can work towards a clinical trial system that is fair, reliable, and transparent, ultimately improving patient outcomes. Therefore, to investigate the reasons for trial discontinuation and nonpublication within orthopaedic surgery, our primary objectives are to: (1) determine the rate of trial discontinuation and nonpublication of trial results among upper and lower extremity fracture RCTs; and (2) identify factors associated with trial discontinuation and nonpublication.

## Materials and methods

### Objective and outcome measures

This investigation employed a cross-sectional study design. We assessed the rate of discontinuation and nonpublication of phase 3 or 4 clinical trials involving human subject participants who had an upper or lower extremity fracture. Data were collected using ClinicalTrials.gov and published trial reports. Human participant data were not used during this investigation; thus, institutional review board oversight was not required per 45 CFR 46.102(d) and (f) of the US Department of Health and Human Services' Code of Federal Regulations [19].

### Identifying eligible orthopaedic clinical trials

We performed a systematic search of registered clinical trials using ClinicalTrials.gov––an online clinical trial repository managed by the United States National Library of Medicine. The decision to use this registry was based on the fact that U.S. clinical trialists are required to: (1) prospectively register their trial on this platform prior to study commencement; and (2) provide regular updates throughout the course of the trial. Each trial is assigned a unique national clinical trial (NCT) number. Data available on each registry includes recruitment status, intervention type, participants, funding, and other relevant data related to the trial. We used the advanced search function on ClinicalTrials.gov using the search terms provided in Additional file 1. ClinicalTrials.gov captures a wide range of potentially relevant articles through use of an automated term mapping system by searching additional, related terms. This search was performed on September 9, 2020.

### Study inclusion and exclusion criteria

We searched for trials that were completed or discontinued by only including trials with the following designations: completed, suspended, terminated, withdrawn, or unknown status. All trials with the completed status were categorized to the “completed” group, and trials with the status terminated, withdrawn, unknown, or suspended were categorized to the “discontinued” group. Trials with the status of active, not recruiting, or enrolling by invitation were excluded. One of us (JMA) screened search results by registered title, condition, study design, and date of completion. Studies were excluded if they were not relevant to upper or lower extremity fractures, were not a Phase 3 or 4 trial, or if the trial was completed after September 9, 2017. We included only Phase 3 and 4 trials because they assess long-term outcomes in diverse human subject participants, with the intent of publication. Studies with multiple phases listed on the ClinicalTrials.gov database were treated as the most advanced phase listed (for example, Phase 2/3 trials were considered phase 3). Additionally, phase 3 or 4 trials are of the highest levels of evidence which likely affects clinical decision making [20–22]. Early phase trials (0–2) are designed for effective dosing, sample size calculations, and intervention safety and are not commonly intended for translation to clinical care [23, 24]. Furthermore, phase 0 and 1 trials are not required to be registered with the CliniclaTrials.gov database [25]. Therefore phase 0, 1, and 2 trials were excluded from our sample and in congruence with previously published methodology [11, 26–28]. The exclusion date of September 9, 2017 was chosen to allow trialists 36 months from the date of study completion for publication, as done in previous studies [18, 29]. The study completion date is when the last participant in a clinical study was examined or received an intervention/treatment to collect final data for the primary outcome measures, secondary outcome measures, and adverse events [30]. To include the largest possible number of RCTs, no limitation was used for patient age or demographics.

### Locating publications of completed trials

To determine the publication status of each included RCT, two investigators (SS, BH) searched for publication information or direct link to the publication on ClinicalTrials.gov. A clinical trial that linked a publication that did not directly report on the results of the trials was not considered a published trial. We only considered a trial as “published” if the trial results were available in the form of a complete manuscript in a peer-reviewed journal. If no publication information was found on ClinicalTrials.gov, two of us (SS, BH) searched MEDLINE via PubMed, Embase, and Google Scholar by trial title, authors, and/or NCT numbers using the same approach. This search algorithm was developed and pilot-tested a priori from a sample of trials in a different field. Our definition of published and our search strategy to locate publications were both adapted from previous studies [18, 31].

### Contacting central contacts

Per ClinicalTrials.gov any questions or inquiries about a specific study should be addressed to the research staff member listed in the trials “Contacts and Study Location” section of the study record. [30, 32] When clinical trials are in the recruiting phase the listed contact is referred to on ClinicalTrial.gov as the “Central Contact” under the contacts and study location tab [30]. We attempted to reach a trial’s central contact for one of the following reasons: (1) if a trial was determined to have not been completed yet failed to provide a reason on ClinicalTrials.gov; and (2) if we were unable to identify a publication using the aforementioned search strategy. To locate the central contacts' email address, we searched ClinicalTrials.gov, institutional websites, Google, and PubMed publications for the central contact. After emails were located, we used a standardized email with several prespecified responses addressing the reason(s) for either discontinuation or nonpublication. Responses to our standardized email were based on a systematic review by Song et al. [33]. We repeated this email strategy once per week for 3 consecutive weeks. If no response was received in 8 weeks, or if the email was returned as inactive, we considered the author as unreachable. This method of email contact has been successfully used in previous studies [18, 29]. Trials were considered to be unpublished if both the search and email strategies failed to reveal evidence of publication.

### Statistical analysis

Descriptive statistics—frequency, percentages, and median (interquartile range, IQR)—for extracted variables were reported. To evaluate the effects of trial characteristic variables on discontinuation and publication status, adjusted odds ratios (AOR) were calculated via multivariable logistic regression. We preselected variables for adjustment of these logistic regression models a priori. Alpha was set at 0.05 for all analyses, which were conducted using STATA 16.1 (College Station, Texas, USA).

## Results

### Study characteristics

**Fig. 1:**
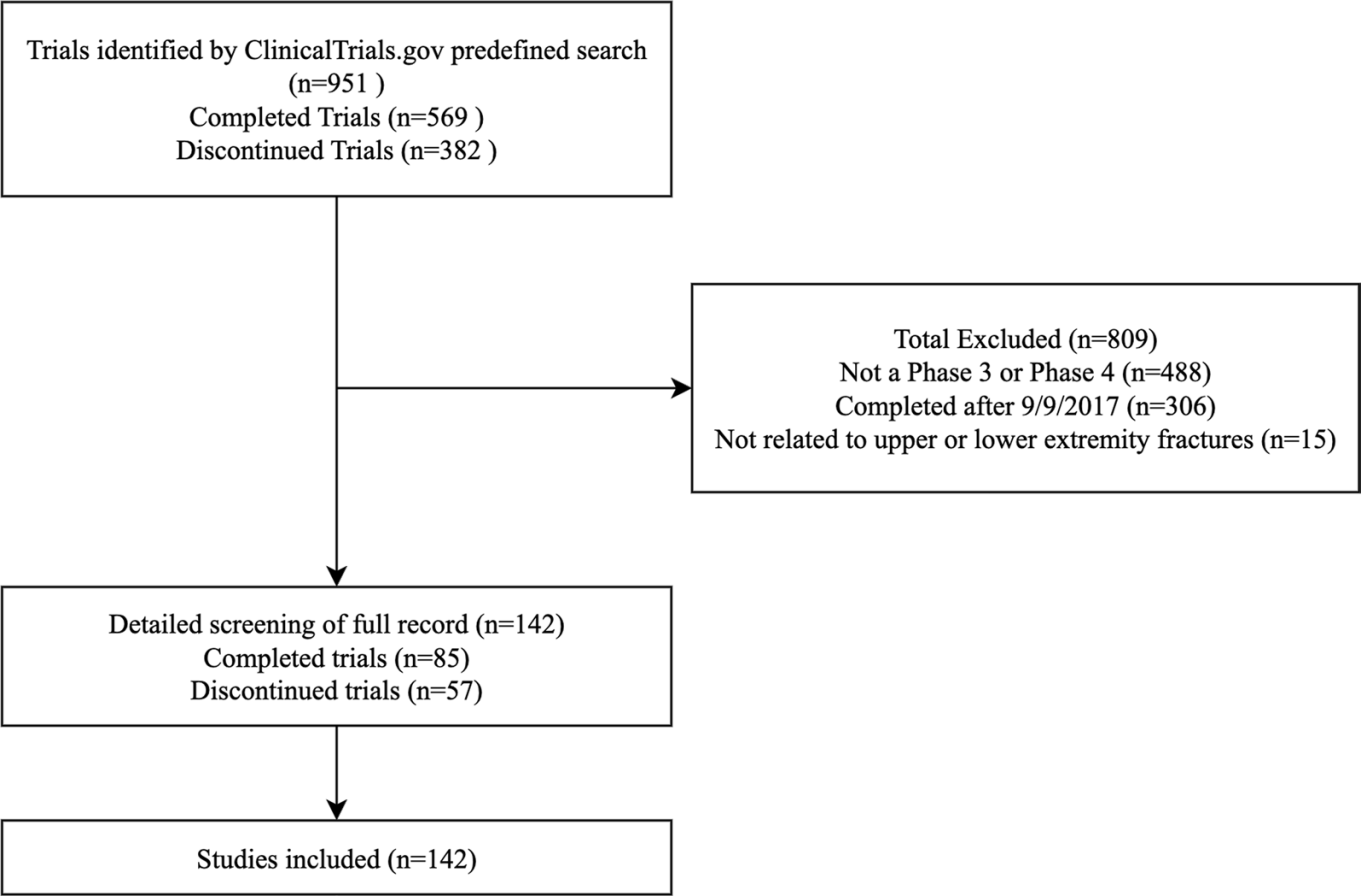
Flow chart for study inclusion and exclusion

Our search of ClinicalTrials.gov resulted in 951 potentially eligible RCTs, of which 142 met the final inclusion criteria (Fig. 1). Seventy-nine (of 142; 55.6%) of the RCTs were classified as phase 4 trials, 52 (of 142; 36.6%) trials were phase 3, and 11 (of 142; 7.7%) were combined phase 2 and 3 trials. All of the trials in our sample were interventional studies, the majority of which investigated pharmaceutical interventions (83/142; 58.5%). The majority of RCTs received hospital (73/142; 51.4%) or industry (30/142; 21.1%) support. The median number of participants enrolled was 81 (IQR = 126) (Table 1). We found that only 29 (of 142; 20.4%) trials in our sample had results linked to ClinicalTrials.gov. The dates of trial registration ranged from September 9th, 1997, to October 31st, 2016.

**Table 1:** Characteristics of completed versus discontinued trials and published versus unpublished trials (*n* = 142)

Characteristic	Total	Trial status			Publication status		
		Discontinued (57)	Completed (85)	*χ*^2^, *P*	Published (71)	Unpublished (71)	*χ*^2^, *P*
*Intervention*							
Pharmaceutical	83 (58.45)	32 (22.54)	51 (35.92)	Pearson *χ*^2^ = 0.44, *P* = .93	46 (32.39)	37 (26.06)	Pearson *χ*^2^ = 2.89, *P* = .41
Behavioral/dietary	6 (4.23)	2 (1.41)	4 (2.82)		2 (1.41)	4 (2.82)	
Device	30 (21.13)	13 (9.15)	17 (11.97)		12 (8.45)	18 (12.68)	
Procedure	23 (16.2)	10 (7.04)	13 (9.15)		11 (7.75)	12 (8.45)	
*Funding*							
Hospital/university	73 (51.41)	31 (21.83)	42 (29.58)	Pearson *χ*^2^ = 6.14, *P* = .11	34 (23.94)	39 (27.46)	Pearson *χ*^2^ = 0.91, *P* = .82
Industry	30 (21.13)	10 (7.04)	20 (14.08)		17 (11.97)	13 (9.15)	
Mixed	29 (20.42)	15 (10.56)	14 (9.86)		15 (10.56)	14 (9.86)	
Other	10 (7.04)	1 (0.7)	9 (6.34)		5 (3.52)	5 (3.52)	
*Published*							
No	71 (50)	45 (78.9)	26 (30.6)	Pearson *χ*^2^ = 31.92, *P* < .001	71 (100)	0 (0.0)	–
Yes	71 (50)	12 (21.1)	59 (69.4)		0 (0.0)	71 (100)	
*Completed*							
No	57 (40.14)	57 (100)	0 (0.0)	–	12 (16.9)	45 (63.4)	Pearson *χ*^2^ = 31.92 *P* < .001
Yes	85 (59.86)	0 (0.0)	85 (100)		59 (83.1)	26 (36.6)	
*Enrollment; Median: 80 (IQR: 37–164)*							
< 80	66 (46.48)	34 (59.6)	32 (37.6)	Pearson *χ*^2^ = 6.64, *P* = .01	24 (33.8)	42 (59.2)	Pearson χ^2^ = 9.17, *P* = .002
≥ 80	76 (53.52)	23 (40.4)	53 (62.4)		47 (66.2)	29 (40.8)	

### Discontinued trials

**Fig. 2:**
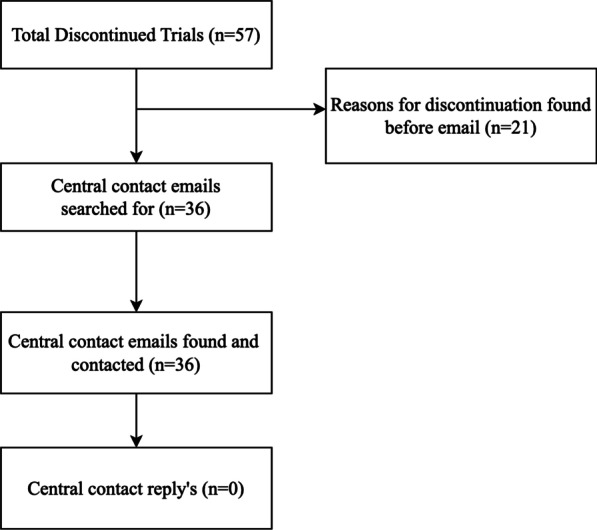
Flow chart for identifying reasons for trial discontinuation

Of the 142 RCTs, 85 (59.9%) were completed and 57 (40.1%) were discontinued. Of the 57 discontinued trials, 24 (42.1%) were terminated, 24 (42.1%) had an “unknown” status, 8 (14.0%) were withdrawn, and 1 (1.8%) was suspended. The registration years for discontinued trials ranged from 1997 to 2016 with no specific year having a markedly higher percentage of discontinued trials. Prior to email 36 RCTs (of 57; 63.2%) did not provide reasons for trial discontinuation on ClinicalTrials.gov. We contacted all 36 primary investigators yet received no reply (Fig. 2). The most common reasons identified for discontinuation on ClinicalTrials.gov included inadequate sample size or recruitment problems (13/21; 61.9%), poor study quality or design (3/21; 14.3%), intervention no longer in production (2/21; 9.5%) withdrawn funding or support from trial sponsor (1/21; 4.8%), adverse events or concerns for patient safety (1/21; 4.8%), and primary investigator left (1/21; 4.8%). Trials with more than 80 participants were less likely to be discontinued (AOR: 0.42; 95% CI 0.2–0.88) compared to trials with fewer than 80 participants (Table 2).

**Table 2:** Logistic regression of trial discontinuation and nonpublication

Characteristic	Discontinued trials (*n* = 57)		Unpublished trials (*n* = 71)	
	No. (%)	AOR (95%CI)	No. (%)	AOR (95%CI)
*Intervention*				
Pharmaceutical	32 (22.54)	1 [Ref]	37 (26.06)	1 [Ref]
Behavioral/dietary	2 (1.41)	1.07 (0.15–7.82)	4 (2.82)	2.8 (0.43–19.37)
Device	13 (9.15)	1.29 (0.52–3.18)	18 (12.68)	1.05 (0.92–5.64)
Procedure	10 (7.04)	1.5 (0.55–4.05)	12 (8.45)	0.78 (0.58–4.17)
*Funding*				
Hospital/university	31 (21.83)	1 [Ref]	39 (27.46)	1 [Ref]
Industry	10 (7.04)	0.96 (0.37–2.51)	13 (9.15)	0.48 (0.38–2.55)
Mixed	15 (10.56)	1.3 (0.53–3.2)	14 (9.86)	0.29 (0.24–1.55)
Other	1 (0.7)	0.14 (0.02–1.26)	5 (3.52)	0.57 (0.18–3.27)
*Enrollment*				
< 80	34 (59.6)	1 [Ref]	42 (59.2)	1 [Ref]
≥ 80	23 (40.4)	**0.42 (0.2–0.88)**	29 (40.8)	**0.12 (0.15–0.66)**

*AOR* adjusted odds ratio

Bold values indicate the Statistical significance set at *P* < 0.05

### Publication status

**Fig. 3:**
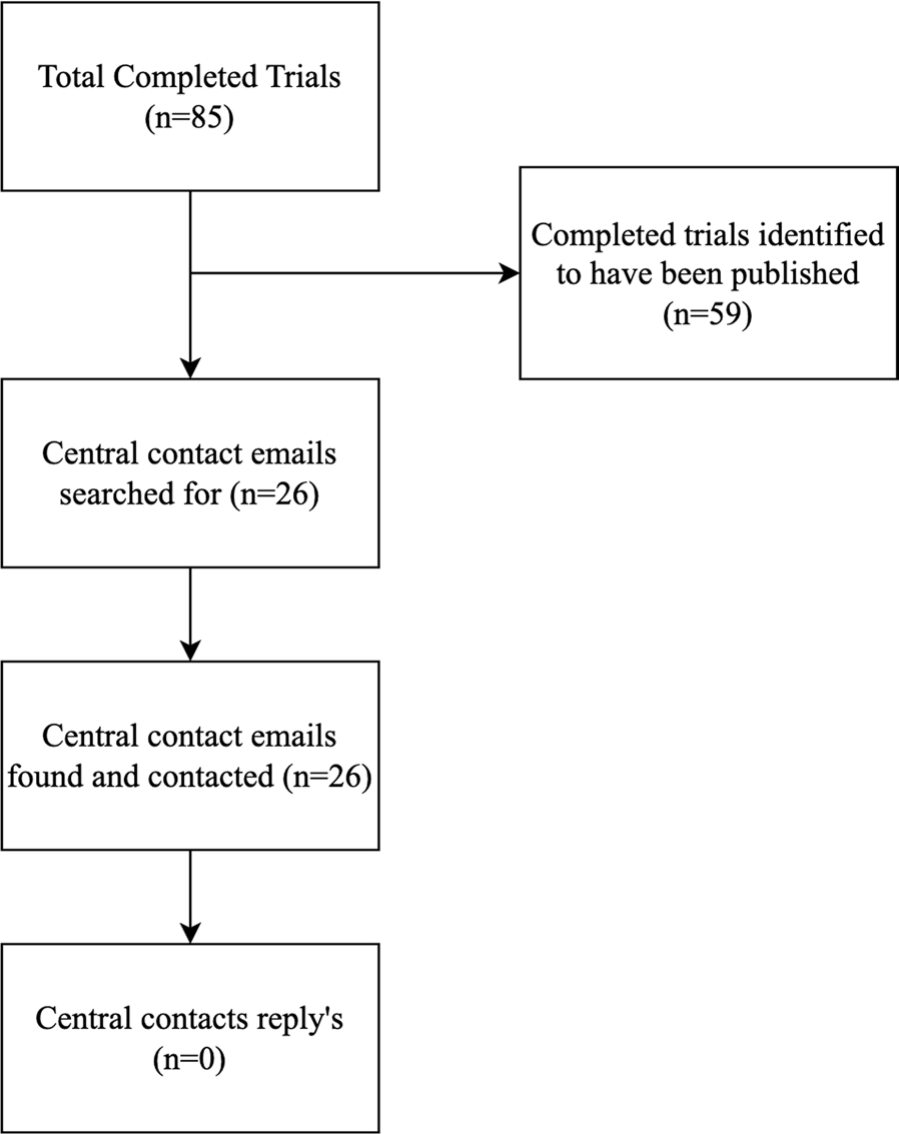
Flow chart for identifying reasons for nonpublication in completed trials

A total of 71 RCTs (of 142; 50.0%) were published in a peer-reviewed journal and 71 RCTs (of 142; 50.0%) were unpublished. Twelve of the 57 discontinued trials (21.1%) reached publication, whereas 59 of the 85 completed trials (69.4%) reached publication. The registration years for unpublished trials ranged from 2000 to 2016 with no specific year having a markedly higher percentage of unpublished trials. Of the 26 completed, yet unpublished, trials, we identified all 26 central contact’s email addresses. However, our effort to determine the status of these unpublished trials was unsuccessful as none of the central contacts replied to our email queries (Fig. 3). Completed trials were more likely to reach publication (59/85; 69.4%; *X*^*2*^ = 31.92; *P* ≤ 0.001) compared to discontinued trials (12/57; 20.1%). Trials with more than 80 participants were found to be less at risk of not reaching publication (AOR: 0.12; 95% CI 0.15–0.66) compared to trials with fewer than 80 participants (Table 2).

## Discussion

Of the 142 upper and lower extremity fracture RCTs included in our analysis, approximately one-half failed to reach publication and two-fifths were discontinued prior to trial completion. Of the discontinued RCTs, over 90% did not provide access to trial results. Similarly, the majority of discontinued trials did not provide a reason for discontinuation on ClinicalTrials.gov. When provided, loss of funding and inadequate recruitment represented the most common reasons for trial discontinuation. Lastly, discontinued trials were less likely to reach publication compared to completed trials. Here, we expand on our findings, compare our findings within the broader context of the existing literature on trial discontinuation and nonpublication, and provide recommendations to address these barriers to help increase the transparency and accessibility of trial data in orthopaedic surgery.

Our results––that 40.1% of trials were discontinued––suggest the rates of discontinuation among upper and lower extremity fracture RCTs is high. Among discontinued trials, over one-third failed to provide a reason for discontinuation on ClinicalTrials.gov. The rate of trial discontinuation––and the rate by which trialists provide a justification for discontinuation––is variable across medical specialties. For example, Johnson et al. [18] reported 29.2% of head and neck cancer clinical trials were discontinued, with 50% of trials failing to provide a reason for discontinuation on ClinicalTrials.gov. In contrast, a 2018 analysis of trials related to osteoarthritis found reasons for discontinuation for 97% of discontinued RCTs [29]. While discontinuation may be problematic in some circumstances, it can be necessary in others [34]. For example, trials may be appropriately discontinued when new evidence supports trial futility, when the benefit of the treatment is superior, and when the risks outweigh the benefits [35]. Randomized controlled trials are also discontinued for preventable reasons [34]. One trial in our sample listed an “adverse event” as the primary reason for discontinuation, however, other trials listed potentially preventable reasons such as inadequate study design, poor recruitment, and lack of funding support [26, 36–39]. Addressing these reasons for discontinuation may help to reduce medical research waste and improve the likelihood of trials reaching publication [40].

We found that completed trials were more likely to reach publication compared to discontinued trials––a finding that is consistent with previous investigations [18, 29]. Publication of trials through a peer-review process has traditionally served as the primary means for disseminating results to the scientific community [9]. However, one-half of the included trials from our sample remain unpublished, thereby hindering the public’s access to these trials’ results. This lack of readily accessible data is compounded by the fact that only one-fifth of included trials provided access to trial results on ClinicalTrials.gov. Failure to provide access to unpublished trial results on a public repository precludes investigators and clinicians from synthesizing these results when attempting to answer clinical questions. In fact, previous researchers have noted that published trials often report larger effect estimates favoring the investigational product compared to unpublished trials [41–43]. Others have reported the exclusion of unpublished trial results from systematic reviews and meta-analyses may lead to an increased likelihood of reporting favorable results, as well as larger overall, yet less precise, effect estimates [41–43]. Taken together, these findings highlight the importance that results from clinical trials have on the research community, regardless of the nature of the findings themselves. Therefore, we contend that clinical trialists, at a minimum, should strongly consider updating the trial’s registry with the most up-to-date and accurate results both during the course of and upon completion or discontinuation of the trial. Doing so will help increase the transparency and validity of ongoing and future research within the field of orthopaedic surgery.

Efforts to mitigate trial discontinuation and nonpublication may serve to increase the accessibility of trial results, regardless of the significance of trial outcomes. Because a large portion of trials within our sample were discontinued for reasons related to trial recruitment and participant retention, we contend that clinical trialists should first direct their attention to developing safeguards against trial discontinuation for seemingly preventable reasons. More specifically, we suggest trialists provide evidence of tested and robust methodology––including how adequate target sample sizes will be obtained, acceptable recruitment periods, and participating site locations. In a 2021 study published in *BMJ Open*, Axen and colleagues highlight common challenges researchers face during the recruitment of study participants––including the relevance of the research question towards potential study subjects, sufficient communication between parties regarding the nature of the study and reassurance of anonymity, time constraints, and trust in the research team [44]. To help address these common challenges, Axen et al. [44] provide a stepwise checklist to help ensure adequate recruitment in pragmatic intervention studies. Implementation of a similar checklist as part of an a priori protocol might help identify gaps in recruitment efforts prior to initiation of the trial, thereby allowing corrections to be made with ample time such that the integrity of the study is not jeopardized. In the event a trial is discontinued, trialists should be required to publish trial results on ClinicalTrials.gov in an appropriate amount of time after trial completion or discontinuation. Currently, the U.S. Food and Drug Administration are making efforts to increase the rate of data sharing on the ClinicalTrials.gov platform by notifying trial sponsors when they are overdue on posting their trial results [45]. Similar efforts from other influential stakeholders in the research community may be a step in the right direction towards decreasing the large proportion of unavailable evidence from orthopaedic clinical trials. Lastly, we encourage orthopaedic journals to follow the lead of journals across other medical specialties by dedicating a section of their journals for null or negative trial results [46, 47]. For example, the American Heart Association journals have developed an online “Null Hypothesis Section” which seeks to promote access to negative, inconclusive, or confirmatory research [48]. A similar initiative in orthopaedic journals would be a major step towards promoting transparency of ongoing research within the field.

Although we attempted to retrieve a maximum of registered trials regarding upper and lower extremity fractures, it is possible some were omitted from our search. Moreover, our sample only included phase 3 and 4 trials and therefore may not be generalizable to other clinical trial phases. It should also be noted that the low response rate to our email queries for unpublished trials could be due to factors such as a change of institution (and therefore the trials central contact email address) without an appropriate update to records on ClinicalTrials.gov [18, 26, 29]. Lastly, our study focused on upper and lower extremity fractures and may not be generalizable to the breadth of orthopaedic literature.

## Conclusion

Rates of discontinuation and nonpublication among upper and lower extremity fracture RCT are high. Discontinuation and nonpublication of clinical trials may subject participants to potentially harmful interventions, limit the advancement of clinical research, and contribute to research waste. On the other hand, some clinical trials are discontinued or not published when the evidence is deemed to be futile, the benefit of the treatment is superior, or when the risk outweighs the benefit. Efforts to mitigate these inconsistent research practices are warranted to improve clinical trials within the field of orthopaedic surgery.

## Supplementary Information

**Additional file 1**. Clinical Trials.gov search criteria

## Data Availability

The datasets used and/or analyzed during the current study are available from the corresponding author on reasonable request.
