# Supplementary material for: Rates of discontinuation and non-publication of upper and lower extremity fracture clinical trials
Source: J Orthop Surg Res. 2023 Mar 29;18:256. doi: 10.1186/s13018-023-03698-5 (PMC10053675; doi:10.1186/s13018-023-03698-5)
Supplement: Supplementary file 1 — Additional file 1. Clinical Trials.gov search criteria [file 13018_2023_3698_MOESM1_ESM.docx]

**Supplemental File 1.** Criteria used to search ClinicalTrials.gov for Upper and Lower Extremity Randomized Clinical Trials

951 records identified through 09/09/2020:

**Condition or disease:** “leg fracture” OR “arm fracture” OR “ankle fracture” OR “elbow fracture” OR “hip fracture” OR “knee fracture” OR “shoulder fracture” OR “forearm fracture” OR “upper extremity fracture” OR “lower extremity fracture” OR “clavicle fracture” OR “clavicular fracture” OR “humerus fracture” OR “humeral shaft fracture” OR “olecranon fracture” OR “ulna fracture” OR “ulnar fracture” OR “radius fracture” OR “radial fracture” OR “both bone forearm fracture” OR “hip fracture” OR “femoral head fracture” OR “femoral neck fracture” OR “intertrochanteric fracture” OR “subtrochanteric fracture” OR “femur fracture” OR “femoral shaft fracture” OR “distal femur fracture” OR “tibia fracture” OR “tibial plateau fracture” OR “tibial shaft fracture” OR “fibula fracture” OR “fibular shaft fracture” OR “fibular head fracture” OR “bimalleolar fracture” OR “trimalleolar fracture” OR “pilon” OR “long bones” OR “wrist fracture” OR “hand fracture” OR “foot fracture” OR “metatarsal fracture” OR “tarsal fracture” OR “metacarpal fracture” OR “carpal fracture” OR “metaphysis fracture” OR “diaphysis fracture” OR “epiphysis fracture” OR “extra-articular” OR “intra-articular”

**Study Type:** “Interventional Studies”

**Trial Status:** “Completed,” “Suspended,” “Terminated,” “Withdrawn,” or “Unknown status”
